# The potential therapeutic effect of platelet-rich plasma in the treatment of post-COVID-19 parosmia

**DOI:** 10.1186/s43163-022-00320-z

**Published:** 2022-10-12

**Authors:** Heba A. Abo El Naga, Reham S. El Zaiat, Ahmad M. Hamdan

**Affiliations:** 1 Otolaryngology Head & Neck Surgery Department, Faculty of Medicine, Menoufia University, Shebin El-kom, Egypt; 2 Clinical Pathology Department, Faculty of Medicine, Menoufia University, Shebin El-kom, Egypt

**Keywords:** Parosmia, COVID-19, Olfactory dysfunction, Platelet-rich plasma

## Abstract

**Background:**

COVID-19-related olfactory dysfunction is an emerging problem with a significant impact on the quality of life of affected individuals. Different lines of treatment have been used with varying results. This study aimed to assess the potential therapeutic effect of PRP in the treatment of post-COVID olfactory dysfunction. This work aimed to assess the potential therapeutic effect of platelet-rich plasma (PRP) in treating post-COVID-19 parosmia. A pilot study was conducted on 60 patients with post-COVID parosmia without responding to a 3-month course of olfactory training, topical corticosteroids, omega-three, vitamin B12, and zinc supplementation. The patients were distributed randomly and equally among 2 groups. The case group was subjected to three PRP injections in the olfactory cleft at 3 weeks intervals. The control group continued the pre-study treatment protocol for 6 weeks. The degree of parosmia was assessed before and after treatment subjectively using a visual analog scale (VAS) from 0 to 10. Reaching 0–1 on the visual analog scale was a complete improvement. The primary outcome was assessing the post-treatment score for parosmia 1 month after the third injection in the case group. The second outcome was the comparison between both groups regarding the degree of improvement 1 month after cessation of treatment.

**Results:**

There was a highly significant improvement in VAS for parosmia (*p* < 0.00001) in the case group and a significant improvement in VAS for parosmia in the control group (*p* = *P* = 0.00148). There was a significant difference between both groups regarding the degree of improvement favoring the case group (*p* = 0.002).

**Conclusion:**

Platelet-rich plasma injection in the olfactory cleft offers a therapeutic option for treating patients with post-COVID-19 olfactory parosmia who failed to respond to traditional conservative treatment.

## Background

Olfactory dysfunction caused by COVID-19 is characterized by a fast onset of impairment, which may come with or without other symptoms. Italian COVID-19 hospitalized patients of young age and female gender were more likely to have a compromised sense of taste or smell [1]. Unpublished studies and personal accounts suggest that olfactory issues can be resolved in 2 weeks or less. However, it is unknown how many people would experience chronic postinfectious olfactory impairment due to a lack of long-term follow-up [2].

Angiotensin-converting enzyme 2 receptor, a necessary component for SARS-CoV-2 entry, is abundant in nasal epithelial cells, making coronaviruses one of several pathogenic organisms that can cause postinfectious olfactory dysfunction [3]. If cells in the olfactory neuroepithelium are disrupted, inflammatory changes in the olfactory neuroepithelium may impact olfactory receptor neuron function, encourage more olfactory receptor neuron damage, and restrict subsequent neurogenesis. Olfactory impairment from such changes may be temporary or persistent [4].

In patients with olfactory dysfunction, the olfactory neuroepithelium and olfactory filae, peripheral nerve fibers that cross the cribriform plate and enter the nasal cavity, may be exploited as therapeutic targets since they can regenerate. An autologous biologic product called platelet-rich plasma (PRP) is created from freshly drawn whole blood with a high platelet content. One of PRP’s anti-inflammatory and pro-regenerative properties is the upregulation of growth factors such as transforming growth factor, vascular endothelial growth factor, epidermal growth factor, and insulin-like growth factor. It has been used as a safe and efficient treatment for inflammation, wound healing, and peripheral neuropathies in various therapeutic settings [5]. PRP has been demonstrated to help with neurodegeneration and axon regeneration. GFs and stem cells have been used in animal experiments to treat anosmia and regenerate olfactory neurons when neurodegenerative processes are present [6, 7]. Due to the high concentration of GFs and neurotrophic factors in PRP, several authors have examined PRP’s effectiveness in treating anosmia through its role in accelerating the healing process in animal models [8]. This study aimed to assess the potential therapeutic effect of PRP in the treatment of post-COVID parosmia.

### Patients and methods

The current study was a pilot study conducted on 60 patients with post-COVID parosmia recruited from the outpatient clinic of the Otorhinolaryngology Department, [Removed for blinding] during the period from December 2020 to December 2021. Approval from the institutional review board was obtained, and informed written consent was taken from every patient before participation in the study.

The inclusion criteria of this study included an age of more than 18 years with a history of COVID-19 infection as confirmed by a polymerase chain reaction (PCR) test more than 3 months before. Patients in the study should have olfactory dysfunction in the form of parosmia without a response to a 3-month course of olfactory training, topical corticosteroids, omega-three, vitamin B12, and zinc supplementation. Patients with active COVID-19 infection, patients with previous nasal surgery, and patients with bleeding tendencies were excluded from the study.

The study patients were randomly distributed among two groups using the block randomization method. The case group included 30 patients subjected to three PRP injections in the olfactory cleft at 3 weeks intervals. The control group included 30 patients who continued the pre-study treatment protocol, including olfactory training, topical corticosteroids, omega-three, vitamin B12, and zinc supplementation for 6 weeks.

The study patients were subjected to an assessment protocol, including history taking to assess the inclusion and exclusion criteria. Complete otorhinolaryngology examination, including endoscopic examination of the nose and computed tomography of the nose and paranasal sinuses, were performed to exclude other intranasal pathologies. The degree of parosmia was assessed subjectively using a visual analog scale from 0 to 10. Reaching 0–1 on the visual analog scale was a complete improvement. The assessment of the degree of parosmia using VAS will be repeated 1 month after cessation of the treatment in each group.

#### Platelet-rich plasma preparation

The procedures outlined by Perez et al. [9] were performed on each patient. [1] After sterilization, 8.5 ml of whole blood was aspirated by venipuncture from the cubital vein using a wide-pore canula, and 1.5 ml of acid citrate dextrose was added (ACD). [2] Neither before nor during platelet separation was the blood cooled. [3] To provide a “soft” spin, the collected tubes on the ACD were centrifuged at 800 rpm for 10 min. [4] Sterile syringes were used to transfer platelet-containing supernatant plasma into another clean, sterile tube (without anticoagulant). [5] To obtain a platelet concentrate, the tubes holding the separated plasma were centrifuged at a higher speed of 2000 rpm (a hard spin). The lower 1/3 of the tubes contained PRP, whereas the top 2/3 contained platelet-poor plasma (PPP). Platelet pellets develop at the tube’s bottom. [6] Platelet pellets were suspended in a minimum amount of plasma (1–2 ml) by gently shaking the tube after PPP was extracted using sterile syringes. [7] To ensure adequate platelet yielding, the platelet count was assessed following PRP preparation.

#### PRP Administration

The PRP administration started with applying a local anesthetic solution prepared by mixing decongestant nasal drops with Emla® 5% and 10% Xylocaine spray and inserted in the nose on cotton pieces for 30 min. With the aid of a nasal endoscope, PRP is injected into the olfactory region approximately every 1 cm^2^ using a 1-ml syringe and 30-G needle.

#### Outcomes

The study’s primary outcome was assessing the post-treatment score for parosmia 1 month after the third injection in the case group. The second outcome was the comparison between both groups regarding the degree of improvement 1 month after cessation of treatment.

#### Statistical analysis

Data were collected, tabulated, and statistically analyzed using an IBM personal computer with Statistical Package of Social Science (SPSS) version 22, IBM Corp, Armonk, NY, USA. Descriptive statistics for quantitative data were presented as mean (¯X) and standard deviation (SD). Qualitative data were presented as numbers and percentages (%). Data turned up to be non-normally distributed according to the Kolmogorov–Smirnov test. Mann–Whitney *U* test was used to compare the quantitative data of both groups. The chi-square test (*χ*^2^) was used to study the association between two qualitative variables. Wilcoxon sign rank test was used to compare both groups' pretreatment and post-treatment scores. A two-sided *p*-value of (≤ 0.05) was considered statistically significant.

## Results

The study included 60 patients with post-COVID-19 parosmia distributed as two similar case and control groups. There was no statically significant difference between the two groups regarding age, gender, and VAS for parosmia (*p* = 0.303, 0.584, and 0.484, respectively) (Table 1).

**Table 1:** Sociodemographic and clinical data of the studied patients

Parameter		Case group		Control group		Statistical test	*p*-value
		**Mean**	**SD**	**Mean**	**SD**	**Mann–Whitney *U* test**	
**Age**		28.9	6.31	30.07	5.74	*Z* = − 1.03491	0.303
**VAS for parosmia**		9.13	0.73	9.27	0.78	*Z* = 0.70226	0.484
		**No**	**%**	**No**	**%**	**Chi-square test**	
**Gender**	**Male**	11	36.7	9	30	*X*^2^ = 0.3	0.584
	**Female**	19	63.3	21	70		

*VAS* Visual analog scale

There was a highly significant improvement in VAS for parosmia (*p* < 0.00001) in the case group and a significant improvement in VAS for parosmia in the control group (*p* = *P* = 0.00148) (Table 2). There was a significant difference between both groups regarding the degree of improvement favoring the case group (*p* = 0.002) (Table 3).

**Table 2:** Comparison between pretreatment and post-treatment VAS for olfactory parosmia in both groups

Group	Pretreatment VAS Mean ± SD	Post-treatment VAS Mean ± SD	Wilcoxon sign rank test	*p*-value
Case group (30 patients)	9.13 ± 0.73	3.33 ± 3.29	*z* = − 4.3724	*p* < 0.00001
Control group (30 patients)	9.27 ± 0.78	7.43 ± 2.84	*z* = − 3.0594	0.002

*VAS* visual analog scale

**Table 3:** Comparison between both groups regarding the degree of improvement of parosmia

Variable		Case group (30 patients)		Control group (30 patients)		Chi-square test	*p*-value
		No	%	No	%		
**Degree of improvement**	**No improvement**	5	16.7	18	56.7	Chi = 15.4005	0.0005
	**Partial improvement**	10	33.3	9	33.3		
	**Complete improvement**	15	50	3	10		

## Discussion

The pathophysiology of COVID-19-induced olfactory impairment has been explained by three theories, including the following: [1] mechanical obstruction brought on by inflammation around the olfactory cleft, which prevents odorants from binding to olfactory receptors [10]; [2] infection of ACE-2 expressing supporting cells, particularly the sustentacular cells of the olfactory epithelium [11]; and [3] direct invasion of olfactory neurons by SARS-COV-2 which prevents the olfactory nerve transmission [12]. Based on the second and third hypotheses, platelet-rich plasma might be a viable approach for treating refractory olfactory impairment.

The basal cells that make up the olfactory epithelium’s basal layer are capable of regeneration [13]. Progenitor cells come in two varieties: globose basal cells (GBC) and horizontal basal cells (HBC) (GBC). GBCs are constantly active and aid in the regeneration of olfactory epithelium cells, whereas HBCs are typically inactive and multiply after lesions. Therefore, activating HBC should aid in improving the performance of the olfactory system [14–18]. In recent years, several studies have been conducted to assess the use of growth factors for activating HBCs in the olfactory epithelium. By triggering olfactory nerve regeneration, statins, for instance, improved degenerative anosmia. Reduced inflammation and the activation of genes linked to cell growth and neurogenesis, which led to cell proliferation and neuroregeneration, were the two ways the olfactory epithelium improved. Basic fibroblast growth factor (bFGF) intranasal treatment has been advantageous. bFGF is a multifunctional growth factor that prevents the death of nerve cells and encourages neuronal sprouting, which might lead to the regeneration of the olfactory epithelium. PRP contains large amounts of platelet-derived growth factor (PDGF), insulin-like growth factor (IGF), neurotrophin-3, angiopoietin-1, and other GFs and neurotropic factors. Its administration is therefore anticipated to have a therapeutic and neuro-regenerative effect, and it may be used as a stimulant of basal cell regeneration in treating anosmia [19–23]. Earlier studies have shown that PRP may activate and create new olfactory system receptors [8].

Platelet-rich plasma has been shown to have neuro-regenerative properties in both peripheral and central nervous system injuries. PRP promotes axon regeneration and recovery of neurological functions after injury of the peripheral nerves through three mechanisms: [1] transform the fibrin within the nerve gap from a passive supporter to an active promoter of nerve axon regeneration [24, 25], [2] direct promotion of nerve axon regeneration [26], [3] promote raging the Schwann cells of the central and distal segments of the nerve to proliferate and upregulate their synthesis and release of neurotrophic and other growth factors that act synchronously with platelet-released factors to promote axon regeneration [27–29], and [4] enhance mesenchymal stem cells differentiation into Schwann cells and release of axon regeneration-promoting factors [30–33].

PRP enhances the central nervous system’s axons regeneration. In organ culture, peripheral axons transform into spinal cord tissue, which would not occur naturally [34]. IGF-1 and VEGF activities play a part in this effect [34]. Thus, PRP can potentially promote axon regeneration and neurological recovery following spinal cord injury. It may also be a therapeutic approach for CNS diseases like traumatic brain injury, autoimmune diseases, and neurodegenerative disorders like Alzheimer’s disease, Parkinson’s disease, and amyotrophic lateral sclerosis [35].

In the current study, there was a highly significant improvement of post-COVID olfactory parosmia following three three-weekly PRP injections in the olfactory cleft with a significant difference compared with the control group favoring the case group. Reviewing the literature, some authors have assessed the role of platelet-rich plasma in treating anosmia without specification of post-COVID olfactory dysfunction. Five patients with anosmia received platelet-rich plasma injections in a study by Mavrogeni et al. [36]. Four patients reported that “their smell came back” after the third and, ultimately, the fourth therapy. In contrast, the last patient claimed he could smell “a lot, but not everything.” The authors claimed that administering platelet-rich plasma to the olfactory area would be a promising, last-ditch treatment for total anosmia. In a pilot study, Yan et al. [37] examined the effectiveness of platelet-rich plasma in the treatment of olfactory dysfunction in seven patients who had an olfactory loss that had lasted longer than 6 months, showed no signs of sinonasal inflammatory disease, and did not improve with olfactory training or topical budesonide rinses. They discovered that all patients initially experienced a subjective improvement in smell following injection but that this improvement quickly stabilized. Two patients with functional anosmia did not significantly improve 3 months after treatment. At the 3-month follow-up, five patients with hyposmia exhibited improvement, with 60% achieving normosmia. Forty-eight anosmia patients with sinonasal polyposis participated in randomized controlled research by Goljanian Tabrizi et al. [38] to evaluate the effectiveness of platelet-rich plasma as an adjuvant therapy to endoscopic sinus surgery. Their research found that PRP injection had no immediate impact on patients with sinonasal polyposis’ ability to regain their olfactory function. Eighty patients with various anosmia causes participated in a prospective trial by Aboelmagd et al. [39] utilizing platelet-rich plasma. The authors found that whereas 34 out of 80 patients (42.5%) exhibited no improvement, 46 out of 80 patients (57.5%) said that “their smell came back.” All patients with idiopathic anosmia showed improvement, although there was no statistically significant difference between the patient and control groups.

The limitations of our study included the lack of objective assessment of olfactory functions using electroolfactogram due to its nonavailability. Although the validated tools for olfactory assessment like the University of Pennsylvania Identification test or Sniffin stick test have been widely used for olfactory assessment in various studies, they presented a challenge in assessing parosmia cases due to the variation of the distorted smells between different parosmia patients. So, the authors used a visual analog scale to assess the magnitude of the problem as described by the patients. The power of this study lies in its role in the primary evaluation of platelet-rich plasma as a treatment for a significant problem affecting the quality of life of a considerable number of patients reaching up to emaciation due to the perceived bad odors of many dietary products.

## Conclusion

Platelet-rich plasma injection in the olfactory cleft offers a therapeutic option for treating patients with post-COVID-19 olfactory parosmias who failed to respond to traditional conservative treatment. Further larger studies are needed to confirm the findings of this study with emphasis on the long-term results of this treatment line.

## Data Availability

The datasets used and/or analyzed during the current study are available from the corresponding author on reasonable request.

## Abbreviations

ACD: Acid citrate dextrose
CNS: Central nervous system
COVID-19: Coronavirus disease of 2019
GBC: Globose basal cells
HBC: Horizontal basal cells
IGF: Insulin-like growth factor
PCR: Polymerase chain reaction
PPP: Platelet-poor plasma
PRP: Platelet-rich plasma
SARS: Severe acute respiratory distress syndrome
VAS: Visual analog scale
